# Supplementary material for: Characterisation of Neurospheres-Derived Cells from Human Olfactory Epithelium
Source: Cells. 2021 Jul 4;10(7):1690. doi: 10.3390/cells10071690 (PMC8307784; doi:10.3390/cells10071690)
Supplement: Supplementary file 1 [file cells-10-01690-s001.zip › cells-1262105 supplementary revised back/Supplementary Files_1st revision.pdf]

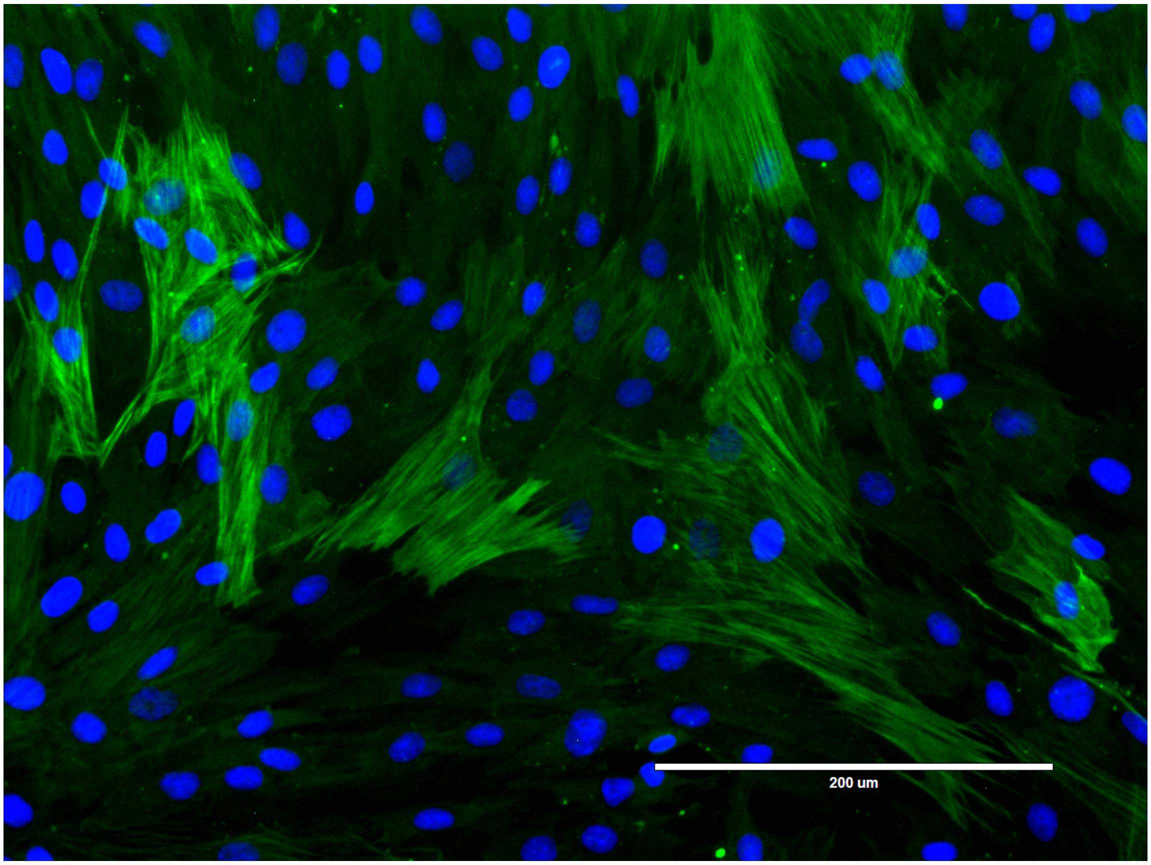

Figure S1. Heterogeneity of SMA in NDC. NDC were stained with SMA (green) and DAPI (blue).

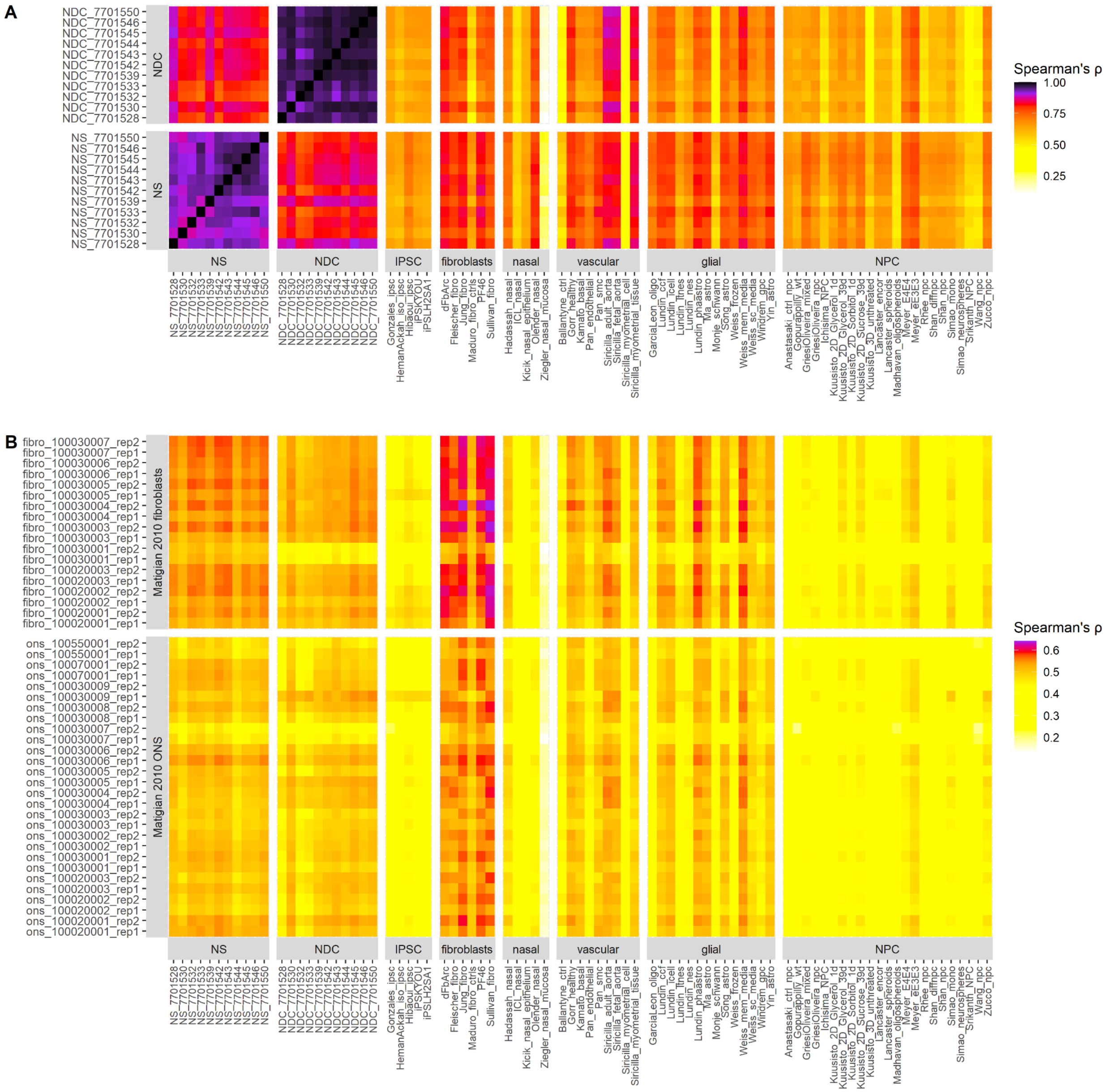

Figure S2. Matrix of Spearman's correlations between analysed expression profiles. (**A**). Spearman rank correlation matrix between NS and NDC expression data with each other and other transcriptomes used as reference, listed in the Supplementary Table S1. Data were prepared as specified in the Methods. (**B**). Spearman rank correlation matrix between Matigian et al. 2010 array expression data and NS, NDC and other transcriptomes used as reference. The Matigian et al. 2010 data were acquired from the ArrayExpress database (accession number E-TABM-724). Files with processed data were used. For the analysis the Illumina probes with unambiguous Ensembl gene IDs were used, the signals for one gene from multiple probes and technical replicates were averaged. Genes outside of the top 90% quantile signal for 90% of probes were filtered out. The intersection of genes (n=1944) with RNAseq data prepared as described earlier was used in the analysis.

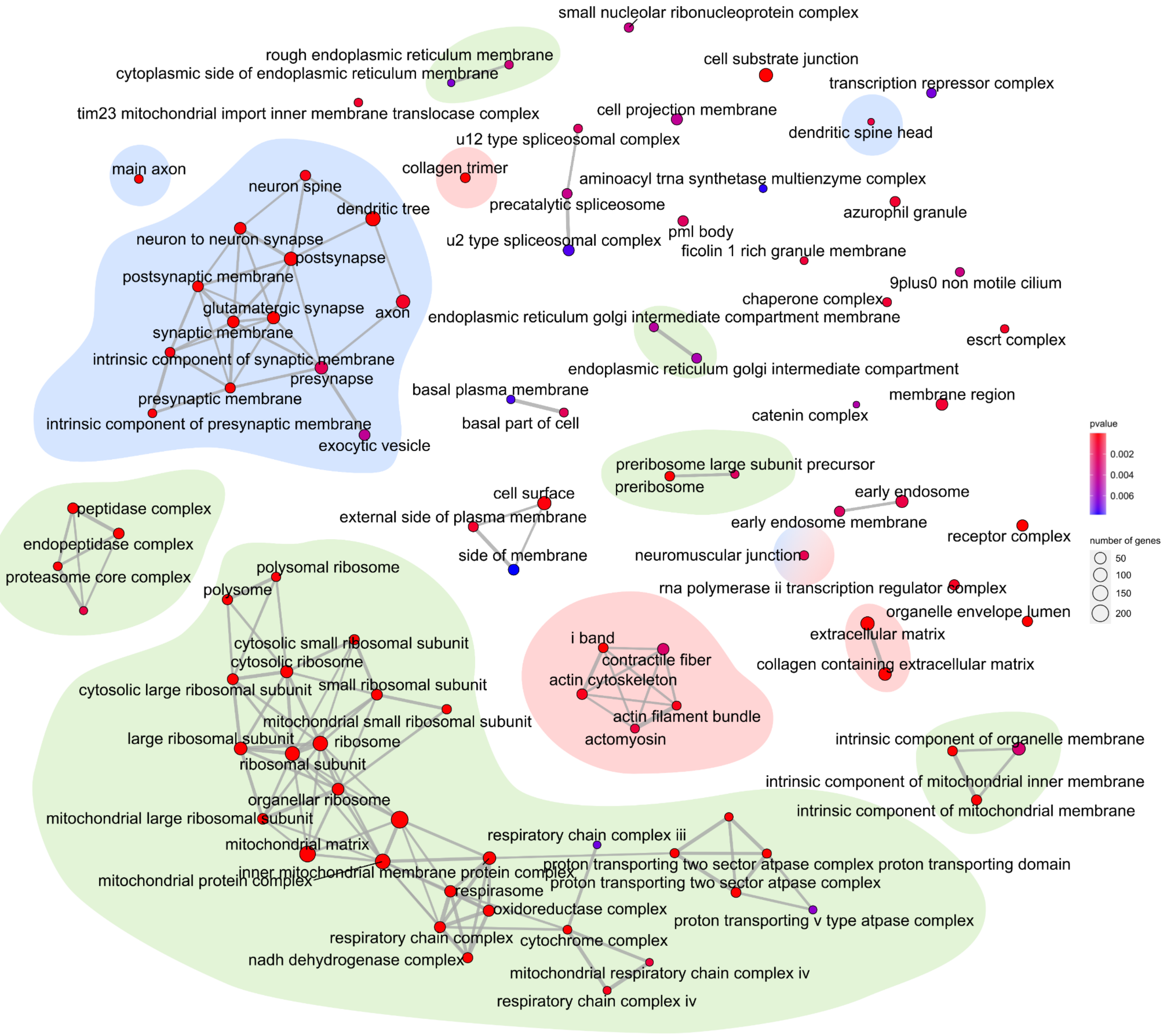

Figure S3. Map of mutually overlapping gene sets, enriched in GSEA analysis of gene ontologies of cellular components (GO:CC). The colored areas represent groups of enriched ontologies: structures involved in cell metabolism, like mitochondria and proteasome (green), neuronal structures (blue) and cytoskeleton and extracellular matrix (red). The colour corresponds to the p-value; the size of the node is proportional to the size of the gene set. Not every node is signed due to lack of drawing space, the complete list of enriched ontologies could be found in Supplementary Table S3.

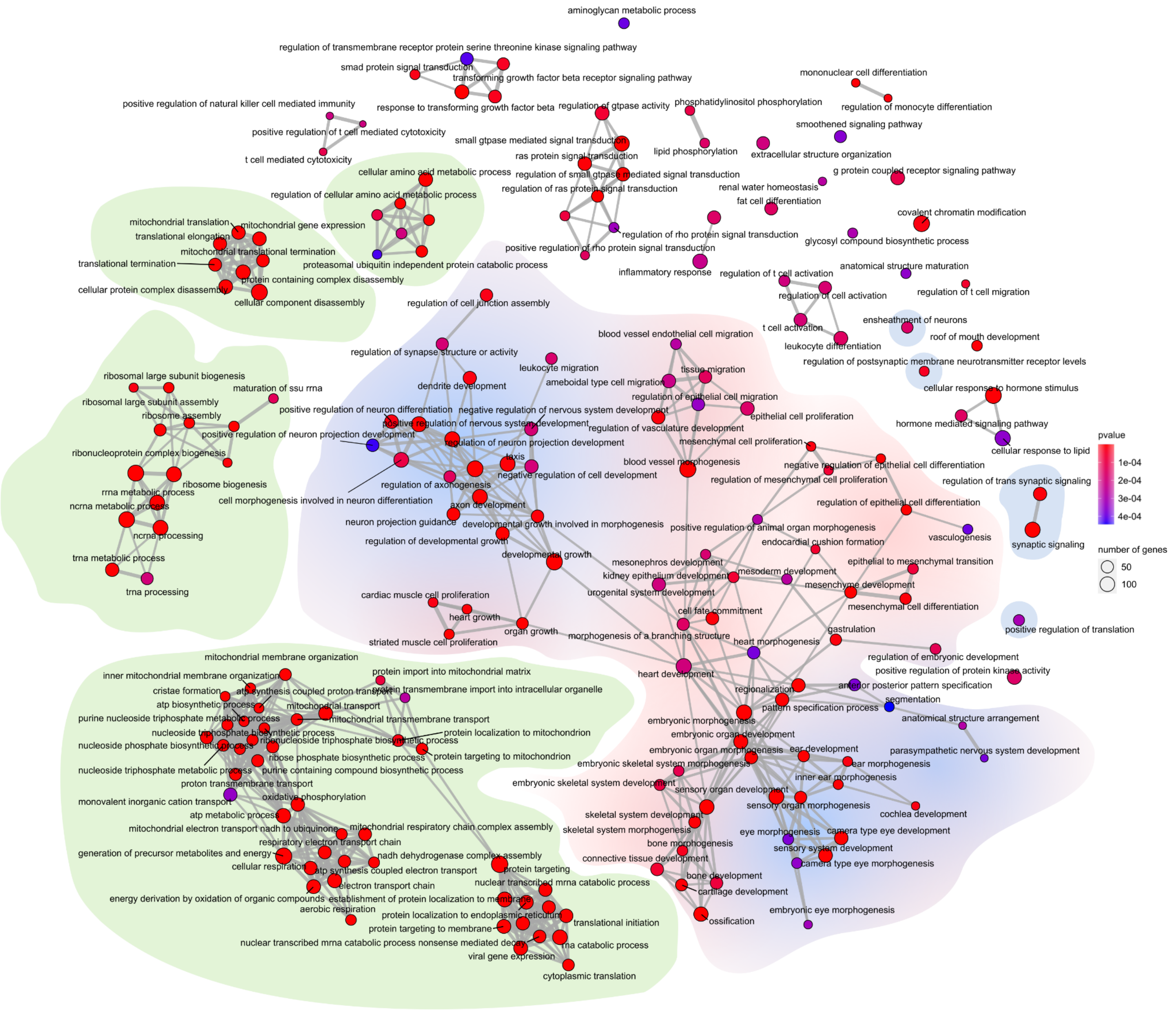

Figure S4. Graph of mutually overlapping gene sets, enriched in GSEA analysis of gene ontologies of biological processes (GO:BP). The colored areas represent three major groups of enriched ontologies: metabolic (green), neuronal and sensory (blue) and non-neuronal, mainly vascular and stromal cell development (red). The most expansive set of ontologies is dyed with blue and red gradients since it could be assigned to both neuronal and non-neuronal areas. The colour of nodes corresponds to the p-value; the size of the node is proportional to the size of the gene set. Not every node is signed due to lack of drawing space, the complete list of enriched ontologies could be found in Supplementary Table S4.

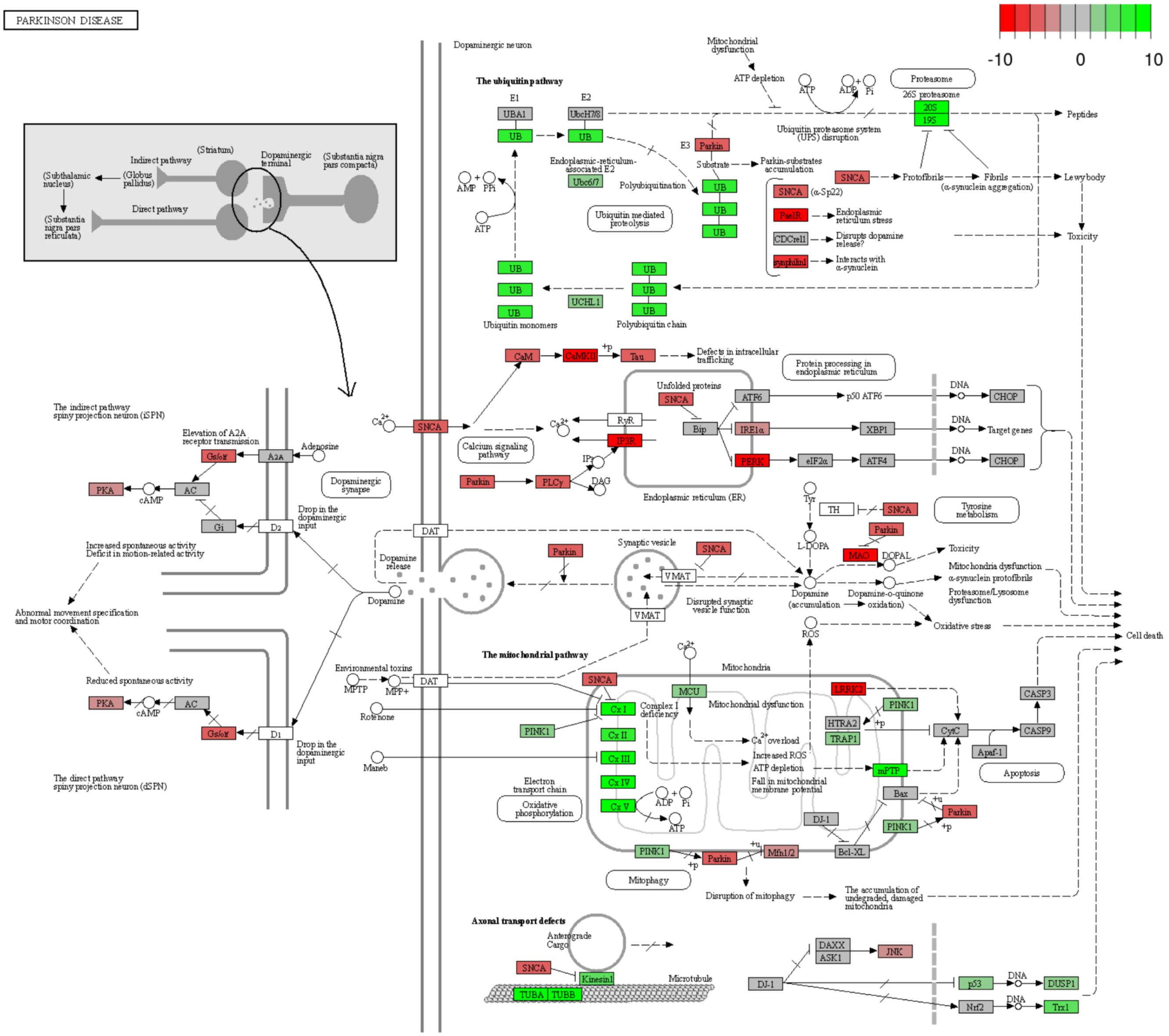

Figure S5. Pathview visualisation of fold change (NDC vs NS) of genes involved in Parkinson's disease. The shades of coloured boxes represent fold change of underlying genes in NDC compared to NS. Green corresponds to upregulated genes, red to downregulated ones.
